# Association Between Interlimb Asymmetry in Normalized Vastus Medialis sEMG Amplitude and Functional Mobility in Patients with Post-Traumatic Knee Contracture: A Cross-Sectional Study

**DOI:** 10.3390/healthcare14162583

**Published:** 2026-08-17

**Authors:** Xiu-Li Kan, Lei-Lei Ji, Qian Lu, Zun-Yu Du, Kai Li, Yong-Zhao Wang, Bin Xiong, Run Zhang, Quan-Bing Zhang, Yun Zhou

**Affiliations:** Department of Rehabilitation Medicine, The Second Affiliated Hospital of Anhui Medical University, Hefei 230601, China

**Keywords:** post-traumatic knee contracture, vastus medialis, surface electromyography, interlimb asymmetry, functional mobility

## Abstract

**Background/Objectives**: Patients with post-traumatic knee contracture often present with limited range of motion (ROM) and impaired functional mobility, but functional performance may not be fully explained by ROM and pain alone. This study aimed to examine whether normalized vastus medialis (VM) surface electromyographic (sEMG) amplitude and its exploratory interlimb ratio were associated with objective functional mobility and patient-reported knee function. **Methods**: This single-center cross-sectional study included 80 patients with post-traumatic knee contracture. Assessments included passive total range of motion (ROM), activity-related pain assessed using the visual analog scale (VAS), Timed Up and Go (TUG) performance, the Simplified Chinese version of the International Knee Documentation Committee Subjective Knee Form (SC-IKDC-SKF), and bilateral VM sEMG amplitude during active knee extension. VM sEMG amplitude was normalized to side-specific maximal voluntary isometric contraction (MVIC) and expressed as %MVIC; the exploratory interlimb ratio was calculated as the affected-side normalized VM sEMG amplitude divided by the unaffected-side value × 100. Spearman correlation, multivariable linear regression, and exploratory hierarchical regression analyses were performed. **Results**: Affected-side normalized VM sEMG amplitude was significantly lower than unaffected-side amplitude (23.8 ± 3.7% vs. 34.0 ± 4.7%, *p* < 0.001), with a mean exploratory interlimb ratio of 70.2 ± 8.8%. In the adjusted regression model, total ROM, activity-related VAS pain score, time to first rehabilitation intervention, and the exploratory interlimb ratio were associated with TUG time; for the interlimb ratio, standardized β = −0.247 and *p* = 0.004. In contrast, SC-IKDC-SKF score was independently associated only with total ROM and activity-related VAS pain score. In the exploratory hierarchical analysis, addition of the interlimb ratio increased the explained variance in TUG time by 4.8% (ΔR^2^ = 0.048, *p* = 0.004) but did not increase the explained variance in the SC-IKDC-SKF score (ΔR^2^ < 0.001, *p* = 0.891). **Conclusions**: Patients with post-traumatic knee contracture demonstrated lower affected-side normalized VM sEMG amplitude and interlimb asymmetry. A lower exploratory interlimb ratio was associated with longer TUG time and increased the explained variance by 4.8% in the exploratory hierarchical analysis, but was not associated with the SC-IKDC-SKF score.

## 1. Introduction

Post-traumatic knee contracture is a common functional complication following trauma to the knee or surrounding structures, fracture fixation, ligament reconstruction, or prolonged immobilization. It typically manifests as reduced knee flexion and/or extension, resulting in impaired mobility and limitations in daily activities. The underlying mechanisms are multifactorial. Intra-articular adhesions, capsular contracture, and soft-tissue restrictions are frequently observed in this condition [1]. Prolonged immobilization, intra-articular fibrosis, and periarticular soft-tissue contracture may further limit knee motion [2]. Fibrotic changes have also been shown to be an important pathological substrate of reduced joint mobility [3], while patellofemoral restriction, intra-articular mechanical block, postoperative scar adhesions, and trauma-induced joint capsule fibrosis may contribute to persistent functional limitation and clinical stiffness [1,2,4].

Although restoration of range of motion (ROM) is a major goal in the management of knee contracture, ROM alone does not fully describe how well a patient moves. In clinical practice, patients with similar ROM may show different levels of difficulty when rising from a chair, walking, turning, or sitting down. Such variability suggests that objective mobility may also be associated with pain, disease duration, immobilization, rehabilitation timing, and neuromuscular factors. Therefore, conventional assessment based on ROM, pain intensity, or patient-reported questionnaires may not adequately characterize differences in functional performance among patients with post-traumatic knee contracture.

Neuromuscular dysfunction has been increasingly recognized as a factor that may contribute to incomplete recovery after knee injury. Studies in anterior cruciate ligament (ACL) reconstruction populations have shown that quadriceps strength, muscle size, voluntary activation, and neuromuscular asymmetry are related to physical function and patient-reported outcomes [5,6]. A systematic review also suggested that knee neuromuscular function may not fully normalize after injury [7]. These measures represent constructs distinct from the normalized sEMG amplitude assessed in the present study and are cited only as indirect clinical context. The vastus medialis (VM) may be particularly relevant because of its role in knee extension and patellofemoral control. Altered VM recruitment has been observed after ACL injury using surface electromyography [8].

However, the relationship between normalized VM sEMG amplitude and functional mobility in post-traumatic knee contracture remains unclear. Most available evidence on neuromuscular impairment comes from ACL-related populations, and it is uncertain whether similar patterns of normalized VM sEMG amplitude are present in patients whose main clinical problem is contracture and restricted motion. To address this gap, the present study normalized VM sEMG amplitude during active knee extension, expressed as a percentage of the side-specific maximal voluntary isometric contraction (%MVIC), and calculated an exploratory interlimb ratio by dividing the affected-side value by the unaffected-side value and multiplying by 100. Because post-traumatic knee contracture typically affects one knee, assessment of the affected side alone may not fully characterize side-to-side differences in normalized VM sEMG amplitude. The exploratory interlimb ratio was examined in relation to the TUG because this test requires bilateral lower-limb contribution during transfers, walking, and turning. However, the ratio was not interpreted as a direct measure of bilateral loading, movement strategy, muscle strength, voluntary activation, arthrogenic muscle inhibition, or overall neuromuscular control. We aimed to examine whether normalized VM sEMG amplitude and its exploratory interlimb ratio were associated with functional mobility and patient-reported knee function. We hypothesized that lower affected-side normalized VM sEMG amplitude and a lower exploratory interlimb ratio would be associated with poorer functional outcomes.

## 2. Materials and Methods

### 2.1. Study Design

This single-center, cross-sectional observational study included patients with post-traumatic knee contracture following knee or periarticular trauma, fracture fixation, ligament reconstruction, or prolonged immobilization. Participant recruitment and data collection were conducted from January to December 2025 after ethical approval had been obtained. At the initial assessment, demographic characteristics, injury- and rehabilitation-related information were recorded. Passive knee ROM, activity-related pain intensity, TUG performance, patient-reported knee function using the Simplified Chinese version of the International Knee Documentation Committee Subjective Knee Form (SC-IKDC-SKF), and bilateral VM sEMG amplitude were then assessed. The study was designed and reported in accordance with the Strengthening the Reporting of Observational Studies in Epidemiology (STROBE) statement [9].

### 2.2. Participants

The inclusion criteria were as follows: (1) age between 18 and 75 years; (2) a clinically diagnosed unilateral post-traumatic knee contracture following knee or periarticular trauma, fracture fixation, ligament reconstruction, or prolonged immobilization, with a persistent and measurable limitation of passive ROM in the affected knee; (3) ability to understand the study procedures and complete the required assessments; and (4) provision of written informed consent. No prespecified numerical cutoff for knee flexion, extension deficit, or passive total ROM was used as an eligibility criterion.

The exclusion criteria were as follows: (1) acute infection, osteoarticular tuberculosis, tumor, or active inflammatory arthropathy; (2) unstable fracture involving the knee joint; (3) severe cognitive impairment or cardiopulmonary disease that precluded completion of rehabilitation assessments; (4) severe bilateral knee ROM limitation; and (5) skin damage, infection, severe scarring, or previous skin grafting at the electrode placement site that could interfere with surface electromyography.

### 2.3. Sample Size Estimation

The sample size was estimated based on the expected correlation between the exploratory interlimb ratio of normalized VM sEMG amplitude and TUG time. Because no directly applicable published effect estimate was available for this specific association in patients with post-traumatic knee contracture, a correlation coefficient of r = 0.35 was used as a pragmatic moderate planning assumption. Assuming a two-sided α of 0.05, a statistical power of 0.80, and an expected correlation coefficient of r = 0.35, at least 62 participants were required. To account for a potential 10–15% rate of missing data, poor sEMG signal quality, or inability to complete functional testing, the target sample size was set at no fewer than 75 participants. Based on patient availability and the feasibility of study implementation at our center, approximately 80 participants were planned for inclusion. Because the final analyses included eight-predictor multivariable and hierarchical regression models, a minimum-detectable-effect sensitivity analysis was additionally performed. With 80 participants, the detectable overall and incremental effects were approximately Cohen’s f^2^ = 0.21 and f^2^ = 0.10, respectively, at α = 0.05 and 80% power.

### 2.4. Demographic and Clinical Data Collection

At enrollment, demographic and clinical data—including age, sex, body mass index (BMI), affected side, injury type, surgical status, disease duration, immobilization duration, and time to first rehabilitation intervention—were recorded. Pain intensity, ROM, TUG performance, SC-IKDC-SKF score, and bilateral VM sEMG were then assessed.

Time to first rehabilitation intervention was defined as the interval from injury or surgery to the first standardized rehabilitation assessment or treatment, expressed in weeks. For patients who did not undergo surgery, the date of injury was used as the starting point, whereas for those who underwent surgery, the date of surgery was used. Because the starting point differed according to surgical status, this variable did not represent an equivalent post-injury interval across all participants.

### 2.5. Pain Assessment

Activity-related pain in the affected knee during the preceding week was assessed using the visual analog scale (VAS), with 0 indicating no pain and 10 indicating the worst imaginable pain [10]. The activity-related VAS score was used as the primary pain measure.

### 2.6. Knee Range of Motion Measurement

The ROM of the affected knee was measured using a GemRed digital goniometer (model 82311-00; GemRed, Guilin, China) [11,12]. Primary ROM measures included passive knee flexion, passive knee extension deficit, and passive total ROM, with the latter calculated as the passive flexion angle minus the extension deficit and used to represent the overall available passive arc of knee motion in the subsequent analyses. All measurements were performed by a single trained assessor.

### 2.7. Timed Up and Go Test

Functional mobility was assessed using the Timed Up and Go (TUG) test [13,14]. Participants stood from a chair, walked 3 m, turned, returned, and sat down. The test was performed three times, and the mean completion time was used for analysis. Participants who routinely used a single-point cane were permitted to use their habitual device, and no study-specific assistive device was prescribed. Longer TUG times indicated poorer mobility.

### 2.8. Patient-Reported Knee Function

Patient-reported knee function was assessed using the validated Simplified Chinese version of the International Knee Documentation Committee Subjective Knee Form (SC-IKDC-SKF) [15]. Scores were converted to a 0–100 scale, with higher scores indicating fewer symptoms and better knee function. The SC-IKDC-SKF was selected as a standardized measure of knee-related symptoms and patient-perceived function. Because it was not specifically developed or validated for patients with post-traumatic knee contracture, it was interpreted as a general knee-related patient-reported outcome rather than a contracture-specific measure. Questionnaires with missing items that precluded calculation of the total score according to the scoring rules were excluded from the relevant analyses.

### 2.9. Surface Electromyography of the Vastus Medialis

Bilateral vastus medialis sEMG signals were recorded using the XMyoMove-EOW surface electromyography system (Jiangxi Nuocheng Electric Co., Ltd., Nanchang, China). Before electrode placement, hair was removed when necessary, the skin was lightly abraded, and the recording sites were cleaned with 70% ethanol. Bipolar surface electrodes were placed over the muscle belly of the vastus medialis according to SENIAM recommendations [16], aligned with the direction of the muscle fibers, with a center-to-center inter-electrode distance of 20 mm. Signals were sampled at 1200 Hz, band–pass filtered at 20–500 Hz, and processed using a 50-ms moving root mean square (RMS) window. The notch-filter setting, exact reference-electrode location, and quantitative skin–electrode impedance criteria were not documented in the original study records. Electrode placement and sEMG testing were performed by a single trained assessor throughout the study.

Before formal recording, the procedures were explained and demonstrated by the assessor, and each participant completed one practice trial to become familiar with the task requirements. For maximal voluntary isometric knee extension, participants were seated with the hip flexed at approximately 90° and the knee fixed at 60° of flexion. After stabilizing the distal lower leg, participants performed maximal knee extension while maintaining the fixed knee angle. Each side was tested three times, with each contraction lasting approximately 5 s and at least 60 s of rest between trials. The RMS value during the stable plateau phase of each trial, excluding contraction onset and release, was extracted, and the highest RMS value across the three trials was used as the side-specific maximal voluntary isometric contraction (MVIC) reference to represent the highest observed MVIC sEMG amplitude and reduce the influence of a potentially submaximal individual trial. MVIC trial acceptability was assessed based on the presence of a stable plateau and the absence of obvious signal artefacts. Force or torque was not recorded during the MVIC trials, and pain during MVIC testing was not quantified separately. Therefore, no force-, torque-, or other mechanically based criterion was available to confirm that a true maximal effort had been achieved.

For the seated active knee extension task, participants actively extended each knee through its maximal attainable range at a smooth self-selected speed and returned to the starting position. The task was performed without external resistance. Limb-specific angular excursion, task duration, and movement velocity were not quantitatively recorded. Each side was tested three times, and participants were instructed to move smoothly without trunk or upper-limb compensation. For each active knee-extension trial, the mean RMS amplitude from the initiation of knee extension to the maximal attainable extension position was calculated. The average of three technically acceptable trials was used to provide a representative task-related estimate and reduce trial-to-trial fluctuation. Trial acceptability was assessed based on the presence of a stable signal and the absence of evident trunk or upper-limb compensation, electrode detachment, or obvious signal artefacts. Unacceptable trials were repeated after an adequate rest period.

VM sEMG amplitude during active knee extension was normalized to the side-specific MVIC RMS value and expressed as normalized VM sEMG amplitude (%MVIC). This normalization was used to reduce the influence of skin impedance, electrode placement, and interindividual differences in electromyographic amplitude [17,18]. The formula was as follows:

Normalized VM sEMG amplitude (%MVIC) = RMS during active knee extension/RMS during MVIC × 100%

The exploratory interlimb ratio of normalized VM sEMG amplitude was calculated as follows:

Exploratory interlimb ratio (%) = affected-side normalized VM sEMG amplitude/unaffected-side normalized VM sEMG amplitude × 100%

The affected-to-unaffected ratio of normalized VM sEMG amplitude was interpreted as an exploratory measure of interlimb asymmetry rather than a direct measure of muscle strength, voluntary activation, or overall neuromuscular capacity.

### 2.10. Main Outcomes and Explanatory Variables

The primary outcome was TUG time, reflecting objective functional mobility, and the secondary outcome was SC-IKDC-SKF score, reflecting patient-reported knee function. The exploratory interlimb ratio of normalized VM sEMG amplitude was retained as the primary sEMG-derived explanatory variable in accordance with the study hypothesis. Affected-side normalized VM sEMG amplitude and the interlimb ratio were not entered simultaneously because the affected-side amplitude was a component of the ratio and the two variables represented closely related sEMG-derived measures.

### 2.11. Statistical Analysis

All statistical analyses were performed using SPSS version 21.0. Continuous variables are reported as mean ± standard deviation, and categorical variables as frequencies and percentages.

Normality of the paired differences was assessed using the Shapiro–Wilk test. Affected- and unaffected-side normalized VM sEMG amplitudes were compared using a paired *t* test when the paired differences were normally distributed, or the Wilcoxon signed-rank test otherwise. The paired mean difference, 95% confidence interval, and Cohen’s *d_z_* were reported. Exploratory Spearman rank correlation was used to assess associations between clinical variables, normalized VM sEMG measures, and TUG time or SC-IKDC-SKF score. Bootstrap 95% confidence intervals were calculated, and the Benjamini–Hochberg false discovery rate procedure was applied across the 18 correlations; only FDR-adjusted *p* values were reported.

Multiple linear regression models were constructed with TUG time and SC-IKDC-SKF score as dependent variables. Candidate variables were selected based on clinical relevance, previous literature, and the study hypothesis. Because the final model specification was not prospectively registered or formally documented before examination of the data, the multivariable analyses were considered hypothesis-guided and exploratory. Each full model included age, BMI, disease duration, immobilization duration, time to first rehabilitation intervention, passive total ROM, activity-related VAS pain score, and the exploratory interlimb ratio of normalized VM sEMG amplitude. To avoid redundancy and potential collinearity, affected-side normalized VM sEMG amplitude and the exploratory interlimb ratio were not entered simultaneously because the affected-side amplitude was a component of the ratio; the interlimb ratio was retained as the primary sEMG-derived explanatory variable.

Unstandardized coefficients with 95% confidence intervals, standardized β coefficients, squared semi-partial correlations, and predictor-specific variance inflation factors were reported.

Multicollinearity was assessed using the variance inflation factor (VIF), with values < 5 considered to indicate no problematic multicollinearity. Regression assumptions and potentially influential observations were assessed using residual diagnostic plots, the Shapiro–Wilk, Breusch–Pagan, Ramsey RESET, and Durbin–Watson tests, externally studentized residuals, and Cook’s distance. Detailed diagnostic results are provided in Supplementary Table S1.

An exploratory hierarchical regression entered age, BMI, disease duration, immobilization duration, and time to first rehabilitation intervention in Step 1; passive total ROM and activity-related pain in Step 2; and the exploratory interlimb ratio in Step 3. Changes in explained variance (ΔR^2^) and the corresponding F-change statistics were reported. Sensitivity analyses addressed potentially influential observations, assistive-device use, the surgically treated subgroup, and broad injury categories. Detailed results are provided in Supplementary Table S1 for influence-point analyses, Supplementary Table S2 for exclusion of single-point cane users, Supplementary Table S3 for additional adjustment for broad injury categories, and Supplementary Table S4 for the surgically treated subgroup. All analyses were based on complete cases; no missing values were imputed, and both primary regression models included 80 participants. All tests were two-sided, with *p* < 0.05 considered statistically significant.

### 2.12. Ethics

This study was approved by the Institutional Ethics Committee of The Second Affiliated Hospital of Anhui Medical University (approval number: YX024-263; approval date: 6 January 2025). All participants were informed of the study purpose, assessment procedures, and potential risks before enrollment, and all provided written informed consent. The study was conducted in accordance with the Declaration of Helsinki. All data were anonymized before analysis.

## 3. Results

### 3.1. Demographic and Clinical Characteristics

**Figure 1:**
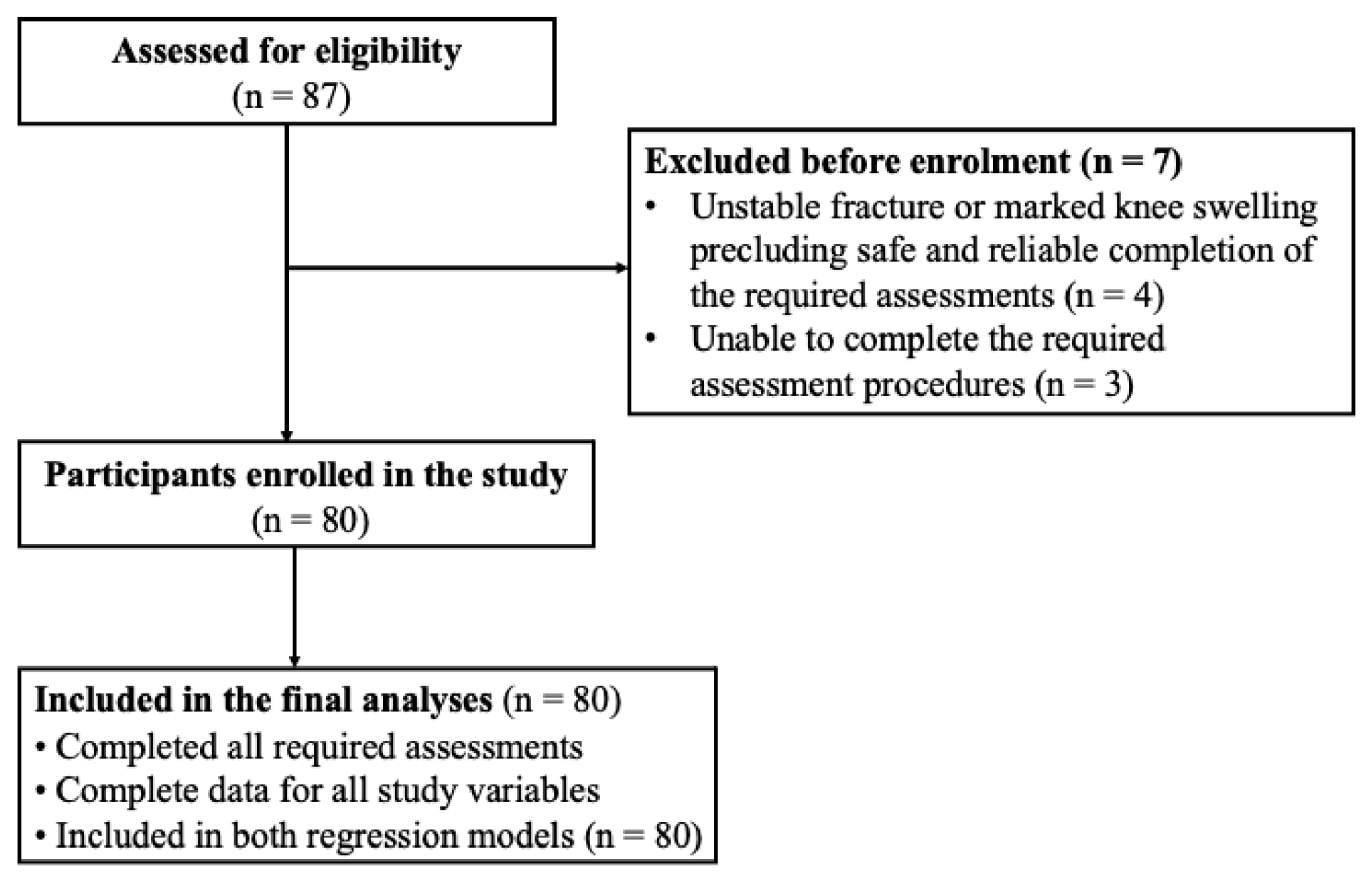
Flow diagram of participant screening, enrolment, and analysis.

A total of 87 patients were assessed for eligibility. Seven were excluded before enrolment: four had unstable fractures or marked knee swelling that precluded safe and reliable completion of the required assessments, and three were unable to complete the required assessment procedures. The remaining 80 participants were enrolled, completed all study assessments, and were included in the final analyses; no participants were lost after enrolment (Figure 1). Complete data were available for all 80 participants for demographic and clinical variables, ROM, activity-related pain, TUG, SC-IKDC-SKF, and bilateral normalized VM sEMG measures. Detailed demographic, clinical, functional, and normalized VM sEMG characteristics are presented in Table 1.

### 3.2. Comparison of Affected- and Unaffected-Side Normalized Vastus Medialis sEMG Amplitudes

Affected-side normalized VM sEMG amplitude was lower than unaffected-side amplitude (23.8 ± 3.7% vs. 34.0 ± 4.7%), with a mean paired difference of −10.24 percentage points (95% CI: −11.03 to −9.44; Cohen’s *d_z_* = −2.86; *p* < 0.001). This represented an approximately 30% lower normalized VM sEMG amplitude on the affected side relative to the unaffected side, and the mean affected-to-unaffected interlimb ratio was 70.2 ± 8.8% (Table 2).

### 3.3. Exploratory Correlation Analysis

After adjustment for multiple testing, longer TUG time was correlated with greater activity-related pain (Spearman’s ρ = 0.603, FDR-adjusted *p* < 0.001), lower passive total ROM (ρ = −0.592, FDR-adjusted *p* < 0.001), lower affected-side normalized VM sEMG amplitude (ρ = −0.272, FDR-adjusted *p* = 0.033), a lower exploratory interlimb ratio of normalized VM sEMG amplitude (ρ = −0.434, FDR-adjusted *p* < 0.001), and a longer time to first rehabilitation intervention (ρ = 0.315, FDR-adjusted *p* = 0.011). Higher SC-IKDC-SKF scores were correlated with lower activity-related pain (ρ = −0.675, FDR-adjusted *p* < 0.001), greater passive total ROM (ρ = 0.644, FDR-adjusted *p* < 0.001), and a higher exploratory interlimb ratio (ρ = 0.324, FDR-adjusted *p* = 0.010). No other correlations were statistically significant after FDR adjustment (Table 3).

### 3.4. Multiple Linear Regression Analysis

Both exploratory eight-predictor models were statistically significant. The TUG model explained 61.8% of the variance in TUG time (adjusted R^2^ = 0.575; F(8, 71) = 14.384; *p* < 0.001). Greater activity-related pain (standardized β = 0.410), lower passive total ROM (β = −0.315), a lower exploratory interlimb ratio of normalized VM sEMG amplitude (β = −0.247), and a longer time to first rehabilitation intervention (β = 0.163) remained associated with longer TUG time. The SC-IKDC-SKF model explained 58.9% of the variance in the score (adjusted R^2^ = 0.542; F(8, 71) = 12.708; *p* < 0.001). Activity-related pain (β = −0.492) and passive total ROM (β = 0.393) remained associated with the SC-IKDC-SKF score, whereas the interlimb ratio was not associated with the score (β = 0.012; *p* = 0.891; Table 4).

Regression diagnostic analyses indicated no major violations of model assumptions. In the exploratory hierarchical analysis, adding the interlimb ratio increased the explained variance in TUG time by 4.8 percentage points (ΔR^2^ = 0.048, F-change = 8.999; *p* = 0.004), but did not increase the explained variance in the SC-IKDC-SKF score (ΔR^2^ < 0.001, F-change = 0.019; *p* = 0.891; Table 5).

In sensitivity analyses, the association between the interlimb ratio and TUG time was attenuated after potentially influential observations were excluded (B = −0.026, 95% CI: −0.053 to 0.001; *p* = 0.056), but remained statistically significant after exclusion of single-point cane users (B = −0.041, 95% CI: −0.071 to −0.011; *p* = 0.008) and after further adjustment for broad injury category (B = −0.042, 95% CI: −0.069 to −0.015; *p* = 0.003). In the surgically treated subgroup, the association between time to first rehabilitation intervention and TUG time was not statistically significant (B = 0.103, 95% CI: −0.027 to 0.232; *p* = 0.118; Supplementary Tables S1–S4).

## 4. Discussion

This cross-sectional study examined whether normalized VM sEMG amplitude and its exploratory interlimb ratio were associated with objective functional mobility and patient-reported knee function in patients with post-traumatic knee contracture. The affected side showed lower normalized VM sEMG amplitude than the unaffected side, indicating interlimb asymmetry. In the full multivariable model, the exploratory interlimb ratio of normalized VM sEMG amplitude was associated with TUG time after adjustment for age, BMI, disease duration, immobilization duration, time to first rehabilitation intervention, total ROM, and activity-related VAS pain score. In the exploratory hierarchical analysis, adding the interlimb ratio increased the explained variance in TUG time by 4.8 percentage points; however, the association was attenuated after potentially influential observations were excluded. In contrast, SC-IKDC-SKF score was mainly associated with total ROM and pain, whereas the exploratory interlimb ratio was not associated with this patient-reported outcome. Overall, the exploratory interlimb ratio was associated with objective mobility performance but not with patient-reported knee function, and these findings should be interpreted cautiously as exploratory cross-sectional associations.

Lower normalized VM sEMG amplitude on the affected side was one of the main sEMG-derived findings of this study. Surface electromyography provides a noninvasive assessment of muscle electrical activity during functional tasks and has been widely used to evaluate task-related muscle activity after knee injury [18]. Evidence from ACL-related populations indicates that quadriceps activation failure, strength deficits, and interlimb neuromuscular asymmetry may persist after reconstruction and are associated with functional outcomes [5,19,20,21]. However, these constructs are distinct from the normalized VM sEMG amplitude measured in the present study, and the ACL-related findings provide only indirect clinical context. Although ACL reconstruction and post-traumatic knee contracture are distinct clinical conditions, evidence specifically examining normalized VM sEMG amplitude in patients with post-traumatic knee contracture remains limited, and findings from ACL-related populations may not be directly generalizable to this population. The lower affected-side normalized VM sEMG amplitude observed in this study therefore indicates a side-to-side difference in task-related VM sEMG amplitude, but should not be interpreted as direct evidence of impaired voluntary activation, muscle weakness, or arthrogenic muscle inhibition. Although the affected-side normalized VM sEMG amplitude was approximately 30% lower than the unaffected-side value, no validated clinically meaningful threshold is currently available for this measure in patients with post-traumatic knee contracture. Therefore, the clinical significance of the observed between-limb difference remains uncertain.

The association between the exploratory interlimb ratio of normalized VM sEMG amplitude and TUG time has a plausible functional context. The TUG integrates sit-to-stand transfer, walking, turning, and return-to-sitting, which require bilateral lower-limb contribution and knee-extensor function. Previous studies in unilateral knee populations have associated quadriceps or weight-bearing asymmetry with task performance and functional mobility [22,23,24,25]. Additional work has associated VM morphology and passive mechanical properties with knee function [26,27]. However, these constructs are distinct from normalized VM sEMG amplitude and provide only indirect clinical context. A lower interlimb ratio in the present study was associated with longer TUG time, suggesting that greater side-to-side differences in normalized VM sEMG amplitude may accompany poorer mobility performance. However, sEMG and bilateral loading were not measured during the TUG; therefore, this interpretation does not establish a directly measured mechanism or causal relationship.

Consistent with the multidimensional nature of the TUG, greater activity-related pain, lower total ROM, a lower exploratory interlimb ratio of normalized VM sEMG amplitude, and a longer time to first rehabilitation intervention remained associated with longer TUG time in the full model. Previous studies have shown that pain and fear of movement or reinjury are related to functional performance during ACL rehabilitation [28], and that arthrogenic muscle inhibition may limit recovery after knee injury [29,30]. Evidence that activation deficits may involve both the injured and uninjured limbs further provides additional indirect context for interpreting interlimb differences [31,32]. However, these constructs were not directly assessed in the present study and should not be regarded as direct validation of the exploratory sEMG-derived ratio.

The different findings for TUG time and SC-IKDC-SKF score may reflect the distinct constructs captured by performance-based tests and self-reported questionnaires. TUG requires coordinated weight bearing, dynamic knee control, balance, and task execution, whereas SC-IKDC-SKF is more closely related to symptoms, perceived activity limitation, and the patient’s own appraisal of knee function. Previous studies have associated quadriceps strength with self-reported function after ACL reconstruction [33], and suggested that quadriceps activation failure may influence the relationship between strength and physical function in knee osteoarthritis [34]. However, muscle strength and activation failure are distinct from normalized VM sEMG amplitude and therefore provide only indirect context for the present findings. In the present study, addition of the exploratory interlimb ratio of normalized VM sEMG amplitude increased the explained variance in TUG time by 4.8 percentage points but did not increase the explained variance in the SC-IKDC-SKF score. This pattern indicates that the ratio was associated with performance-based mobility but not with patient-reported knee function in this sample, without establishing differential clinical utility or incremental validity.

Time to first rehabilitation intervention remained associated with TUG time in the full model. Evidence from ACL and knee rehabilitation studies suggests that muscle atrophy, altered motor control, and delayed neuromuscular recovery may contribute to persistent functional deficits [35,36]. Rehabilitation guidelines also emphasize progressive management of pain and swelling, ROM restoration, strength training, neuromuscular control, and functional task practice [37,38]. Although these data are not specific to post-traumatic knee contracture, they provide only general clinical context for the observed association. However, time to first rehabilitation intervention was calculated from the date of injury in non-surgically managed participants and from the date of surgery in surgically treated participants; therefore, this variable did not represent an equivalent interval across the full sample. In the surgically treated subgroup, the coefficient remained positive and similar in magnitude to that in the full-sample model, but the 95% confidence interval included zero. Accordingly, the full-sample association should be interpreted cautiously and not as evidence that rehabilitation timing directly affected TUG performance, because injury severity, surgical indication, postoperative restrictions, complications, and access to rehabilitation may have contributed to the observed association.

The exploratory interlimb ratio of normalized VM sEMG amplitude should be interpreted with caution. Although it reflects affected-side normalized VM sEMG amplitude relative to the unaffected limb and remained associated with TUG time in the full model, it should not be regarded as a standalone measure of functional capacity or as a direct measure of muscle strength, voluntary activation, or overall neuromuscular capacity. Limb symmetry indices can overestimate knee function when the uninvolved limb also has deficits [39]. In post-traumatic knee contracture, prolonged pain, immobilization, and reduced activity may also affect the unaffected limb. For this reason, the interlimb ratio was analyzed as a continuous exploratory variable rather than using a fixed cutoff to classify interlimb asymmetry as normal or abnormal. This approach avoided imposing an unvalidated threshold whose clinical meaning has not been established in this population. In addition, because each limb was normalized to its own MVIC reference, pain, apprehension, or an inability to generate a true maximal contraction may have influenced the side-specific denominator and the derived ratio.

Clinically, these findings highlight the potential value of a multidimensional approach to functional assessment in patients with post-traumatic knee contracture. ROM describes passive knee-motion limitations, VAS reflects pain burden, TUG provides a performance-based measure of mobility performance, and SC-IKDC-SKF captures patient-perceived symptoms and function. In this context, normalized VM sEMG amplitude and its exploratory interlimb ratio describe side-specific and interlimb sEMG differences, respectively; however, the present findings do not establish their incremental validity, clinical utility, or treatment implications. Because this study did not compare rehabilitation interventions, whether these measures improve clinical assessment or help guide rehabilitation should be evaluated in prospective or interventional studies.

This study has several limitations. Its single-center cross-sectional design precludes causal, temporal, or predictive interpretations, while the broad age range, heterogeneous injury and treatment backgrounds, absence of a prespecified ROM threshold, and modest sample size may limit generalizability and leave residual confounding. Because the original sample-size calculation was based on a bivariate correlation rather than the final multivariable models, smaller independent or incremental associations may not have been reliably detected. Assistive-device use during the TUG was not standardized, the SC-IKDC-SKF was not specifically validated for post-traumatic knee contracture, and only VM sEMG amplitude during active knee extension was assessed. Differences in task mechanics and MVIC effort, together with the absence of formal reliability and measurement-error estimates, may have influenced the normalized sEMG values and derived interlimb ratio. Because the TUG association was attenuated after exclusion of potentially influential observations, the findings should be considered exploratory and confirmed in prospective studies.

## 5. Conclusions

Patients with post-traumatic knee contracture demonstrated lower normalized VM sEMG amplitude on the affected side and interlimb asymmetry. A lower exploratory interlimb ratio of normalized VM sEMG amplitude was associated with longer TUG time after adjustment for relevant clinical variables. In the exploratory hierarchical analysis, addition of the interlimb ratio increased the explained variance in TUG time by 4.8 percentage points but did not increase the explained variance in the SC-IKDC-SKF score. However, this association was attenuated and no longer reached conventional statistical significance after exclusion of potentially influential observations and should therefore be interpreted cautiously. The cross-sectional design does not establish causality, temporal direction, or longitudinal predictive value.

## Figures and Tables

**Table 1:** Demographic and clinical characteristics of the patients.

Variable	Value
Sample size	80
Sex, male/female	36/44
Affected side, left/right	37/43
Injury type, *n* (%)	
Periarticular fracture	34 (42.5)
Ligament injury/reconstruction	30 (37.5)
Soft-tissue injury	7 (8.8)
Meniscus-related injury/surgery	5 (6.3)
Arthroplasty-related contracture	4 (5.0)
Surgical intervention for current injury, *n* (%)	
Yes	73 (91.3)
No	7 (8.8)
Age, years	41.7 ± 15.0
BMI, kg/m^2^	23.5 ± 2.1
Activity-related VAS pain score	4.4 ± 0.9
Passive total ROM, degrees	86.9 ± 10.7
Disease duration, months	2.9 ± 1.6
Immobilization duration, weeks	4.7 ± 1.5
Time to first rehabilitation intervention, weeks	5.6 ± 1.9
TUG time, s	12.5 ± 1.4
SC-IKDC-SKF score	69.4 ± 5.4
Affected-side normalized VM sEMG amplitude, %MVIC	23.8 ± 3.7
Unaffected-side normalized VM sEMG amplitude, %MVIC	34.0 ± 4.7
Exploratory interlimb ratio of normalized VM sEMG amplitude, %	70.2 ± 8.8
Use of a single-point cane during TUG, *n* (%)	11 (13.8)

**Table 2:** Side-specific normalized vastus medialis surface electromyographic amplitudes and the derived exploratory interlimb ratio.

**Panel A. Paired comparison of side-specific normalized VM sEMG amplitude**						
**Measure**	**Affected side**	**Unaffected side**	**Mean difference**	**95% CI**	**Cohen’s *d_z_***	***p***
Normalized VM sEMG amplitude, %MVIC	23.8 ± 3.7	34.0 ± 4.7	−10.24	[−11.03, −9.44]	−2.86	<0.001
**Panel B. Derived Exploratory Interlimb Measure**						
**Measure**						**Value**
Exploratory interlimb ratio of normalized VM sEMG amplitude, %						70.2 ± 8.8

Note. Values are presented as mean ± SD. Normalized VM sEMG amplitude is expressed as a percentage of the side-specific maximal voluntary isometric contraction (%MVIC). The paired mean difference was calculated as affected side minus unaffected side and is reported in percentage points; negative values indicate lower affected-side amplitude. Cohen’s *d_z_* represents the standardized paired difference, and the *p* value was obtained from a paired *t* test. The exploratory interlimb ratio was calculated as affected-side divided by unaffected-side normalized VM sEMG amplitude × 100. As this is a derived within-participant measure without an independent comparator, no separate hypothesis test was performed.

**Table 3:** Exploratory Spearman correlations with TUG time and SC-IKDC-SKF score.

Variable	TUG Time		SC-IKDC-SKF Score	
	ρ (95% CI)	FDR-Adjusted *p*	ρ (95% CI)	FDR-Adjusted *p*
Age, years	0.076 [−0.141, 0.297]	0.751	−0.066 [−0.278, 0.153]	0.773
BMI, kg/m^2^	0.012 [−0.196, 0.219]	0.985	0.010 [−0.204, 0.223]	0.985
Disease duration, months	−0.039 [−0.253, 0.181]	0.936	−0.024 [−0.257, 0.213]	0.985
Immobilization duration, weeks	−0.149 [−0.368, 0.077]	0.337	−0.001 [−0.214, 0.220]	0.993
Time to first rehabilitation intervention, weeks	0.315 [0.107, 0.497]	0.011	−0.172 [−0.387, 0.055]	0.256
Activity-related VAS pain score	0.603 [0.424, 0.737]	<0.001	−0.675 [−0.796, −0.515]	<0.001
Passive total ROM, degrees	−0.592 [−0.718, −0.430]	<0.001	0.644 [0.499, 0.751]	<0.001
Affected-side normalized VM sEMG amplitude, %MVIC	−0.272 [−0.487, −0.029]	0.033	0.117 [−0.116, 0.351]	0.490
Exploratory interlimb ratio of normalized VM sEMG amplitude, %	−0.434 [−0.610, −0.224]	<0.001	0.324 [0.095, 0.527]	0.010

Note. *n* = 80. Values are Spearman’s ρ (percentile bootstrap 95% CI; 10,000 paired resamples). *p* values were adjusted across the 18 exploratory correlations using the Benjamini–Hochberg FDR procedure. FDR, false discovery rate; TUG, Timed Up and Go; VM, vastus medialis; sEMG, surface electromyography; MVIC, maximal voluntary isometric contraction.

**Table 4:** (**a**) Exploratory multivariable linear regression model for TUG time. (**b**) Exploratory multivariable linear regression model for SC-IKDC-SKF score.

(**a**)							
**Variable**	**B**	**SE**	**95% CI**	**β**	***p***	**sr^2^**	**VIF**
Age, years	−0.009	0.008	[−0.024, 0.006]	−0.093	0.237	0.008	1.142
BMI, kg/m^2^	0.041	0.053	[−0.065, 0.146]	0.060	0.448	0.003	1.137
Disease duration, months	−0.034	0.070	[−0.174, 0.107]	−0.036	0.633	0.001	1.078
Immobilization duration, weeks	−0.036	0.075	[−0.185, 0.113]	−0.036	0.631	0.001	1.060
Time to first rehabilitation intervention, weeks	0.125	0.061	[0.004, 0.247]	0.163	0.043	0.023	1.152
Passive total ROM, degrees	−0.042	0.012	[−0.065, −0.019]	−0.315	<0.001	0.072	1.383
Activity-related VAS pain score	0.658	0.138	[0.383, 0.933]	0.410	<0.001	0.122	1.375
Exploratory interlimb ratio of normalized VM sEMG amplitude, %	−0.041	0.014	[−0.068, −0.014]	−0.247	0.004	0.048	1.263
(**b**)							
**Variable**	**B**	**SE**	**95% CI**	**β**	***p***	**sr^2^**	**VIF**
Age, years	0.004	0.029	[−0.055, 0.063]	0.011	0.890	0.000	1.142
BMI, kg/m^2^	0.023	0.208	[−0.393, 0.438]	0.009	0.913	0.000	1.137
Disease duration, months	0.105	0.277	[−0.447, 0.657]	0.030	0.706	0.001	1.078
Immobilization duration, weeks	−0.330	0.294	[−0.916, 0.255]	−0.088	0.265	0.007	1.060
Time to first rehabilitation intervention, weeks	−0.189	0.239	[−0.665, 0.287]	−0.065	0.431	0.004	1.152
Passive total ROM, degrees	0.199	0.045	[0.109, 0.290]	0.393	<0.001	0.112	1.383
Activity-related VAS pain score	−2.986	0.542	[−4.066, −1.906]	−0.492	<0.001	0.176	1.375
Exploratory interlimb ratio of normalized VM sEMG amplitude, %	0.007	0.053	[−0.099, 0.113]	0.012	0.891	0.000	1.263

Model summary for (a): *n* = 80; R^2^ = 0.618; adjusted R^2^ = 0.575; F(8, 71) = 14.384; *p* < 0.001. Model summary for (b): *n* = 80; R^2^ = 0.589; adjusted R^2^ = 0.542; F(8, 71) = 12.708; *p* < 0.001. Note. B, unstandardized regression coefficient; β, standardized regression coefficient; CI, confidence interval; sr^2^, squared semi-partial correlation; VIF, variance inflation factor; ROM, range of motion; VAS, visual analogue scale; VM, vastus medialis; sEMG, surface electromyography; TUG, Timed Up and Go. sr^2^ represents the unique proportion of outcome variance associated with each predictor in the full model. The analyses were exploratory.

**Table 5:** Exploratory hierarchical multivariable linear regression models for TUG time and SC-IKDC-SKF score. (**A**) TUG time, s. (**B**) SC-IKDC-SKF score, points.

(**A**)							
**Step**	**Variables entered**	**R^2^**	**Adjusted R^2^**	**ΔR^2^**	**ΔF**	**df**	***p* for ΔF**
1	Age, BMI, disease duration, immobilization duration, and time to first rehabilitation intervention	0.102	0.041	0.102	1.674	5, 74	0.152
2	Passive total knee ROM and activity-related VAS pain score	0.570	0.528	0.468	39.227	2, 72	<0.001
3	Exploratory interlimb ratio of normalized VM sEMG amplitude	0.618	0.575	0.048	8.999	1, 71	0.004
(**B**)							
**Step**	**Variables entered**	**R^2^**	**Adjusted R^2^**	**ΔR^2^**	**ΔF**	**df**	***p* for ΔF**
1	Age, BMI, disease duration, immobilization duration, and time to first rehabilitation intervention	0.053	−0.011	0.053	0.825	5, 74	0.536
2	Passive total knee ROM and activity-related VAS pain score	0.589	0.549	0.536	46.902	2, 72	<0.001
3	Exploratory interlimb ratio of normalized VM sEMG amplitude	0.589	0.542	<0.001	0.019	1, 71	0.891

Note. *n* = 80. For Step 1, ΔR^2^ and ΔF are relative to the intercept-only model; for Steps 2 and 3, they are relative to the preceding model. ROM, range of motion; VAS, visual analogue scale; VM, vastus medialis; sEMG, surface electromyography; TUG, Timed Up and Go. The analyses were exploratory.

## Data Availability

The participant-level data are not publicly available because of ethical and privacy considerations. De-identified data supporting the findings of this study may be made available from the corresponding author upon reasonable request, subject to applicable ethical and data-protection requirements.

## Supplementary Materials

The following supporting information can be downloaded at: https://www.mdpi.com/article/10.3390/healthcare14162583/s1, Supplementary Table S1, Regression diagnostics and influence-point sensitivity analyses for the primary eight-variable models; Supplementary Table S2, Sensitivity analysis excluding participants who used a single-point cane during the TUG assessment; Supplementary Table S3, Sensitivity analyses additionally adjusting for broad injury categories; Supplementary Table S4, Sensitivity analysis of the TUG regression model restricted to surgically treated participants.
