# Supplementary material for: Association Between Interlimb Asymmetry in Normalized Vastus Medialis sEMG Amplitude and Functional Mobility in Patients with Post-Traumatic Knee Contracture: A Cross-Sectional Study
Source: Healthcare (Basel). 2026 Aug 17;14(16):2583. doi: 10.3390/healthcare14162583 (PMC13512780; doi:10.3390/healthcare14162583)
Supplement: Supplementary file 1 [file healthcare-14-02583-s001.zip › healthcare-4416605-supplementary.pdf]

Supplementary Table S1. Regression diagnostics and influence-point sensitivity analyses for the primary eight-variable models.

Diagnostic assessment	TUG time model	SC-IKDC-SKF score model
Shapiro–Wilk test for residual normality	W = 0.981; P = 0.289	W = 0.984; P = 0.417
Breusch–Pagan test for homoscedasticity	LM = 9.200; P = 0.326	LM = 2.866; P = 0.942
Ramsey RESET test	F = 1.505; P = 0.224	F = 0.191; P = 0.664
Durbin–Watson statistic	2.060	1.869
VIF range	1.060-1.383	1.060-1.383
Maximum absolute externally studentized residual	2.521	2.528
Maximum Cook's distance	0.183	0.161
Observations with Cook's distance > 4/n	3	6
Observations with Cook's distance > 1	0	0
Sensitivity analysis excluding observations with Cook's distance > 4/n	n = 77; ratio B = −0.026; 95% CI [−0.053, 0.001]; P = 0.056	n = 74; ratio B = 0.024; 95% CI [−0.076, 0.125]; P = 0.630

Note. n = 80 in both primary models. The Cook's distance screening threshold was 4/n = 0.050; no observation had Cook's distance > 1. The sensitivity analyses excluded observations above the 4/n threshold. VIF, variance inflation factor; TUG, Timed Up and Go.

Supplementary Table S2. Sensitivity analysis excluding participants who used a single-point cane during the TUG assessment.

Variable	B	SE	95% CI	Standardized β	P	sr²	VIF
Age, years	−0.009	0.008	[−0.024, 0.007]	−0.096	0.261	0.008	1.152
BMI, kg/m²	0.029	0.057	[−0.086, 0.143]	0.042	0.620	0.002	1.159
Disease duration, months	−0.068	0.077	[−0.222, 0.086]	−0.073	0.381	0.005	1.081
Immobilization duration, weeks	0.024	0.081	[−0.138, 0.186]	0.025	0.766	0.001	1.090
Time to first rehabilitation intervention, weeks	0.124	0.065	[−0.006, 0.255]	0.166	0.061	0.023	1.201
Passive total knee ROM, degrees	−0.038	0.012	[−0.063, −0.014]	−0.289	0.002	0.064	1.305
Activity-related VAS pain score, 0-10	0.662	0.146	[0.370, 0.954]	0.417	<0.001	0.129	1.349
Exploratory interlimb ratio of normalized VM sEMG amplitude, %	−0.041	0.015	[−0.071, −0.011]	−0.255	0.008	0.046	1.402

Model summary: n = 69; $R^2 = 0.624$; adjusted $R^2 = 0.574$; $F(8, 60) = 12.446$; $P < 0.001$.

Note. B, unstandardized regression coefficient; β, standardized regression coefficient; CI, confidence interval; sr², squared semi-partial correlation; VIF, variance inflation factor; ROM, range of motion; VAS, visual analogue scale; VM, vastus medialis; sEMG, surface electromyography; TUG, Timed Up and Go. The analysis was exploratory.

Supplementary Table S3. Sensitivity analyses additionally adjusting for broad injury categories.

A. TUG time, s

Variable	B	SE	95% CI	Standardized β	P	sr^2	VIF
Age, years	−0.010	0.008	[−0.026, 0.007]	−0.100	0.256	0.007	1.430
BMI, kg/m²	0.055	0.054	[−0.053, 0.162]	0.080	0.315	0.005	1.175
Disease duration, months	−0.040	0.071	[−0.181, 0.101]	−0.043	0.574	0.002	1.082
Immobilization duration, weeks	−0.023	0.075	[−0.174, 0.127]	−0.024	0.756	0.001	1.078
Time to first rehabilitation intervention, weeks	0.132	0.061	[0.010, 0.254]	0.171	0.035	0.025	1.165
Passive total knee ROM, degrees	−0.043	0.012	[−0.066, −0.020]	−0.318	<0.001	0.073	1.385
Activity-related VAS pain score, 0-10	0.672	0.140	[0.393, 0.952]	0.419	<0.001	0.123	1.423
Exploratory interlimb ratio of normalized VM sEMG amplitude, %	−0.042	0.014	[−0.069, −0.015]	−0.258	0.003	0.052	1.275
Ligament-related injury category	0.266	0.260	[−0.254, 0.785]	0.090	0.311	0.006	1.450
Other injury category	0.385	0.299	[−0.211, 0.981]	0.108	0.202	0.009	1.303

Model summary: n = 80; $R^2 = 0.630$; adjusted $R^2 = 0.576$; $F(10, 69) = 11.732$; $P < 0.001$. Addition of broad injury category: $\Delta R^2 = 0.011$; $\Delta F(2, 69) = 1.047$; $P = 0.357$.

Supplementary Table S3 (continued). Sensitivity analyses additionally adjusting for broad injury categories.

B. SC-IKDC-SKF score, points

Variable	B	SE	95% CI	Standardized β	P	sr^2	VIF
Age, years	0.000	0.033	[−0.067, 0.066]	−0.001	0.990	<0.001	1.430
BMI, kg/m²	0.031	0.215	[−0.397, 0.460]	0.012	0.885	<0.001	1.175
Disease duration, months	0.101	0.281	[−0.460, 0.662]	0.029	0.719	0.001	1.082
Immobilization duration, weeks	−0.328	0.300	[−0.927, 0.271]	−0.088	0.278	0.007	1.078
Time to first rehabilitation intervention, weeks	−0.180	0.243	[−0.665, 0.306]	−0.062	0.462	0.003	1.165
Passive total knee ROM, degrees	0.199	0.046	[0.107, 0.291]	0.392	<0.001	0.111	1.385
Activity-related VAS pain score, 0-10	−3.004	0.558	[−4.119, −1.890]	−0.495	<0.001	0.172	1.423
Exploratory interlimb ratio of normalized VM sEMG amplitude, %	0.007	0.054	[−0.101, 0.115]	0.011	0.899	<0.001	1.275
Ligament-related injury category	−0.054	1.038	[−2.124, 2.016]	−0.005	0.959	<0.001	1.450
Other injury category	0.368	1.191	[−2.007, 2.744]	0.027	0.758	0.001	1.303

Model summary: n = 80; $R^2 = 0.590$; adjusted $R^2 = 0.530$; $F(10, 69) = 9.909$; $P < 0.001$. Addition of broad injury category: $\Delta R^2 < 0.001$; $\Delta F(2, 69) = 0.060$; $P = 0.942$.

Note. Fracture-related conditions were the reference category. The ligament-related indicator represented ligament injury or reconstruction; the other category combined soft-tissue, meniscus-related, and arthroplasty-related conditions. B, unstandardized regression coefficient; β, standardized regression coefficient; CI, confidence interval; sr^2, squared semi-partial correlation; VIF, variance inflation factor; ROM, range of motion; VAS, visual analogue scale; VM, vastus medialis; sEMG, surface electromyography; TUG, Timed Up and Go. The analyses were exploratory.

Supplementary Table S4. Sensitivity analysis of the TUG regression model restricted to surgically treated participants.

Variable	B	SE	95% CI	Standardized β	P	sr²	VIF
Age, years	−0.008	0.008	[−0.025, 0.008]	−0.089	0.303	0.007	1.192
BMI, kg/m²	0.054	0.055	[−0.056, 0.164]	0.083	0.328	0.006	1.160
Disease duration, months	−0.044	0.074	[−0.191, 0.103]	−0.048	0.553	0.002	1.069
Immobilization duration, weeks	−0.044	0.079	[−0.201, 0.114]	−0.044	0.582	0.002	1.056
Time to first rehabilitation intervention, weeks	0.103	0.065	[−0.027, 0.232]	0.136	0.118	0.015	1.205
Passive total knee ROM, degrees	−0.042	0.012	[−0.067, −0.017]	−0.311	0.001	0.069	1.404
Activity-related VAS pain score, 0-10	0.636	0.143	[0.350, 0.922]	0.403	<0.001	0.120	1.354
Exploratory interlimb ratio of normalized VM sEMG amplitude, %	−0.044	0.015	[−0.073, −0.015]	−0.271	0.003	0.056	1.297

Model summary: n = 73; $R^2 = 0.610$; adjusted $R^2 = 0.561$; $F(8, 64) = 12.517$; $P < 0.001$.

Note. In this subgroup, time to first rehabilitation intervention was measured from surgery. B, unstandardized regression coefficient; β, standardized regression coefficient; CI, confidence interval; sr², squared semi-partial correlation; VIF, variance inflation factor; ROM, range of motion; VAS, visual analogue scale; VM, vastus medialis; sEMG, surface electromyography; TUG, Timed Up and Go. The analysis was exploratory.
